# SMOOTHING THE MOVE FROM POST-ACUTE TO HOME CARE FOR OLDER CARDIAC PATIENTS: A SOCIAL WORK TRANSITIONS INITIATIVE

**DOI:** 10.1093/geroni/igz038.3479

**Published:** 2019-11-08

**Authors:** Orah R Burack, Jessica Auerbach-Burgoon, Sandra Mundy, Verena R Cimarolli

**Affiliations:** 1 The New Jewish Home, New York, New York, United States; 2 LeadingAge LTSS Center @UMass Boston, Washington, District of Columbia, United States

## Abstract

Transitioning across medical settings (e.g. from hospital to post-acute (PA) or PA to homecare (HC)) is a difficult time with numerous challenges, as critical information passes across sites, new systems are quickly established, and caretakers change. Older cardiac heart failure (CHF) patients, often with comorbidities and having fewer social supports, are especially vulnerable to rehospitalizations at that time. This study examines the impact of a Social Work Transitions (SWT) intervention, designed to ease older cardiac patients’ transition from a PA to HC setting, on rehospitalization rates. The SWT model for CHF patients was developed in a large healthcare system with a continuum of services for older adults including PA and HC. Once a patient enters PA from the hospital a transitions social worker (SW) remains the patient’s primary support and contact through PA discharge and the transition to HC. In HC, that same SW ensures needed services occur, conducts home visits, and provides additional follow-up via phone calls. Study 1: compared HC rehospitalization rates of CHF patients receiving SWT (N=28) with those not receiving SWT (N=26). This natural control group arose during the initial program months, as SW turnover occurred and some CHF patients were not accompanied by a transitions SW. SWT patients had half the rehospitalizations (25%) as controls (54%). Study 2 tracked 30 day rehospitalizations rates for the first 17 study months (N=257). Program rehospitalization rates (16.7%) were below the CMS benchmark (21%). These findings support using the SWT program to prevent unnecessary rehospitalizations in CHF patient.

